# New directions in evidence-based policy research: a critical analysis of the literature

**DOI:** 10.1186/1478-4505-12-34

**Published:** 2014-07-14

**Authors:** Kathryn Oliver, Theo Lorenc, Simon Innvær

**Affiliations:** 1 School of Social Sciences, University of Manchester, Bridgeford Street, Manchester M13 9PL, UK; 2 Department of Science, Technology, Engineering and Public Policy (STEaPP), University College London, 66-72 Gower Street, London WC1E 6BT, UK; 3 Faculty of Social Sciences, Oslo University College, P.O Box 1084, Blindern, 0317 OSLO, Norway

**Keywords:** Critical analysis, Evidence-based policy, Knowledge utilization, Science and technology studies

## Abstract

Despite 40 years of research into evidence-based policy (EBP) and a continued drive from both policymakers and researchers to increase research uptake in policy, barriers to the use of evidence are persistently identified in the literature. However, it is not clear what explains this persistence – whether they represent real factors, or if they are artefacts of approaches used to study EBP. Based on an updated review, this paper analyses this literature to explain persistent barriers and facilitators. We critically describe the literature in terms of its theoretical underpinnings, definitions of ‘evidence’, methods, and underlying assumptions of research in the field, and aim to illuminate the EBP discourse by comparison with approaches from other fields. Much of the research in this area is theoretically naive, focusing primarily on the uptake of research evidence as opposed to evidence defined more broadly, and privileging academics’ research priorities over those of policymakers. Little empirical data analysing the processes or impact of evidence use in policy is available to inform researchers or decision-makers. EBP research often assumes that policymakers do not use evidence and that more evidence – meaning research evidence – use would benefit policymakers and populations. We argue that these assumptions are unsupported, biasing much of EBP research. The agenda of ‘getting evidence into policy’ has side-lined the empirical description and analysis of how research and policy actually interact *in vivo*. Rather than asking how research evidence can be made more influential, academics should aim to understand what influences and constitutes policy, and produce more critically and theoretically informed studies of decision-making. We question the main assumptions made by EBP researchers, explore the implications of doing so, and propose new directions for EBP research, and health policy.

## Introduction: the evidence-based policy movement

Although sceptics can be found, few researchers or policymakers would publicly disagree that evidence-based policy (EBP) is a goal for both academe and government. Reports describing the importance – and difficulty of – achieving this goal are published with regularity [1-3]. Perhaps unlike other disciplines, EBP has staunch advocates who contribute to an ever-increasing body of commentary around the subject.

EBP is sometimes said to have derived from evidence-based medicine (EBM), which dates back at least to 1972, with Archie Cochrane’s seminal work on effectiveness and efficiency [4]. Since the early 1970s, both practitioners and academics have also considered how policy – in the sense of larger-scale decisions about the delivery and management of services at a population level – could be based on, or informed by, evidence [5,6]. For Cochrane and his heirs, the goal of EBM was to bring about the abandonment of harmful and ineffective interventions, and the adoption of interventions shown to be effective for clinical outcomes. This was to be achieved by implementing findings from systematic reviews and meta-analyses of robust outcome evaluations, ideally randomised trials [7-10].

However, this straightforward narrative of evaluation-based EBP modelled on medicine has long existed in a broader landscape of initiatives to foster closer and more effective links between research and policy (or between researchers and policy-makers). In the UK, for example, these ideas can be traced back at least to the Rothschild experiment, evaluated by Kogan and Henkel in 1983 [11]. The aim of this funding initiative was to enable the health research system to respond to policymakers’ priorities for research [12]. The experiment was largely abandoned, which Kogan and Henkel ascribed to cultural differences between researchers and policymakers, the need for interaction, and other barriers [12,13]. Recent publications, again in the UK, from the Government Office for Science have provided guides for both academics and policymakers who wish to engage with the other group [14,15], and there is increasing interest within academic and higher education bodies generally about how academics may be able to increase their impact, the nature of this impact, and implications of the ‘impact agenda’ [16-18]. However, the tenor of these publications still tends to focus on promoting the use of academic research, rather than studying the practices of knowledge production and policymaking and implementation.

As a result, specific questions about the use of research within policy have become divorced from a broader perspective on policy-making. Many researchers in what may be termed ‘applied research’ fields use terms like ‘knowledge translation’, ‘knowledge exchange’, or ‘evidence use’ without providing clear definitions of what knowledge is part of which decision-making process [19-21]. The result has been a loss of clarity with respect to how evidence is supposed to improve decision-making, what constitutes and defines ‘evidence’ and ‘policy’, and which processes and outcomes are targeted by political or academic efforts. Nevertheless, both EBM and EBP have achieved substantial financial and political support, and substantial weight by the creation of a collection of organisations dedicated to producing evidence-based policy and practice recommendations, guidelines, and best practice statements, such as the *What Works* centres [22]. For health policy and health management in particular, there remains a prominent discourse about moral, ethical, political, and often financial imperatives to use evidence to make the best (value) decisions [23] – without, ideally, disenfranchising non-expert publics [24]. The study of the use of evidence in policy varies from negative to positive advocacy, from simplistic to complex understandings of the processes involved, from uncritical technical approaches to highly cynical commentary. Broadly, practitioners and academics have focused on facets of the use of evidence by policymakers and practitioners, have written polemics encouraging colleagues to do so [23,25-29], identified barriers and facilitators into evidence use [30,31], and designed interventions to increase the use of evidence by policymakers [32]. Evidence-based policy and practice, knowledge translation, and related concepts have become touchstones across a vast range of disciplines – almost sub-disciplines in their own right, with canons and conceptual toolkits of their own.

While much of this work remains mainly theoretical (e.g., [33]), there is a rapidly growing empirical evidence base on barriers and facilitators of evidence use. Some idea of the extent and nature of this literature can be gained by looking at systematic reviews. Three reviews are particularly relevant here [30,31,34]; their methods, findings, and conclusions are summarised in Table 1.

**Table 1:** Comparing three systematic reviews on evidence-based policy

	**Orton, 2011**	**Innvær, 2002**	**Oliver, 2014**
Aims	To synthesise evidence about the extent, types, process of evidence use, and barriers and facilitators	Synthesise facilitators of and barriers to the use of research evidence by health policymakers	To update Innvær 2002 and identify new evidence in this area
Inclusion criteria and search date	Europe, Canada, Australia, and NZ only, must explore “*how research evidence is used in decision-making for public health*”. Primary studies only	Interview studies with health policy decision-makers responsible for decisions on behalf of organisations	All studies reporting barriers or facilitators of use of evidence, from 2002–2011
Types of studies included	18 included, interviews and surveys	24 studies in 26 papers, interviews and surveys	145 included studies: 13 systematic reviews, 42 interviews/qualitative studies, 13/25 entirely/included survey
Types of results presented	PMs perceptions about the use of evidence; qualitative and closed-response	PMs perceptions about the use of evidence; qualitative and closed-response. Types of ‘use’, theories about evidence use	Perceived and observed factors affecting evidence use; definitions of evidence; theories used in included studies
Use of evidence?	Self-reported use of evidence in 2 studies, undermined by quality concerns	21/24 examined actual decision-making processes, all measured perceptions of use or hypothetical use of evidence	33 studies examine research uptake (amount/rate), 50 examine processes of research uptake, 18 examine the impact of research use
Synthesis and QA used	Narrative synthesis; CASP-based QA	Descriptive synthesis; methodological QA	Descriptive synthesis; no QA
Main facilitators	Improved relationships; researchers trained to disseminate, clear, relevant and easy-access research; PM trained in research skills; change of policy culture	Personal contact between researchers and PMs; timeliness and relevance of research, with clear recommendations and high quality; research confirming current policy	Available, clear and relevant research evidence; relationships, collaboration, and contact between researchers and PMs; timing, practical managerial support
Main barriers	Unclear, irrelevant or low-quality evidence. ‘Gulf’ between researchers and policymakers. Lack of PM research skills. Other pressures; practical constraints: financial, time frames, access to research, presentation, and interpretation	Absence of personal contact between researchers and policymakers; lack of timeliness or relevance; mutual mistrust between scientists and policymakers Power and budget struggles	lack of clear or relevant research evidence and costs; lack of timeliness or opportunity; lack of PM research skills or awareness
Theory	None cited	Weiss, Caplan; two-communities thesis	Range of theories, reports which papers used which theory
Conclusions and implications	Action to address the barriers and facilitators needs to be taken; training to overcome barriers to research use; research on interventions to increase research uptake	Studies partially support common beliefs about barriers and facilitators, with little empirical evidence; no strong recommendations about research and policy can be made; limited data support two-communities and Weiss’s theories	Research into managerial and organizational barriers may be more useful than individual-level; relational approach could be used

Common findings across all three include the importance of personal relationships and contacts between decision-makers and researchers, and the need for research to be clearly and accessibly presented. Cultural and practical barriers to the use of evidence by policy-makers are identified. Finally, all three make the point that policymakers’ definitions of evidence do not match academic constructions of ‘evidence’. All three reviews also point to gaps in the literature as priorities for further research, but differ in their identification of these gaps. Orton focuses on the need for evaluation research of strategies to increase the uptake of research evidence [31], while Innvær et al. [34] and Oliver et al. [30] are both more circumspect, pointing out that despite the size of the evidence base, much about policy-makers’ attitudes to research evidence remains unclear. Innvær et al. show how the limited available evidence mainly describes policymakers’ beliefs and attitudes, rather than actual behaviours, and hence cannot be used as a basis to make strong recommendations [34]. Perhaps more importantly, both reviews note that there are few grounds by which to make firm recommendations or conclusions about the process, impact, or effectiveness of research in policy. Only rarely is enough detail known about the policy process to be able to comment usefully: for example, who are the main actors, where are decisions made, and how evidence fits into the process.

The structure of this paper is as follows. First, drawing on a dataset from a recent systematic review of barriers and facilitators [30], we offer a high-level overview of the literature on evidence use in health policy, drawing out broad theoretical and definitional commitments. This paper does not primarily consider the findings of those studies, which are summarised above and set out in more detail elsewhere [30]. We also draw on similar findings about policymakers in non-health fields [35]. We set these data in the context of the wider theoretical literature about evidence and policy, drawing insights from policy sciences. Secondly, we aim to identify and challenge some of the more normative assumptions which are widely prevalent (if often implicit) in the EBP literature, particularly the following: that the policy-evidence ‘gap’ needs ‘bridging’; that policy is usually not based on any data; that policy requires research evidence, preferably evaluative intervention research evidence; and that if more evidence were used in policy making, policy would be better. Finally, we make suggestions for a new agenda for EBP research.

We are not advocating against EBP. On the contrary, we believe that better policy decisions would be a desirable outcome, and that evidence ought to play a role in those decisions. Rather, our conclusions from this critical review of the literature are about EBP research, rather than EBP itself. Researchers have directed their attention at how to increase the impact of their own outputs, rather than on understanding the processes behind policy change. The support for EBP is not as single-minded or vociferous as it was. Reflecting on this historical trend, and reasons behind any such shift, we present our novel contribution to the literature: an illumination of the discourse around EBP by comparing theories, methods, and substantive approaches with those from other fields, and on this basis propose new directions for EBP research.

## Review: evidence-based policy research

Below, we describe some of the underlying concepts and approaches available to researchers attempting to understand the relationship between research and policy processes.

## Theoretical underpinnings

Innvær et al. described how the literature at the time fell into two camps [34]; those supporting the ‘two-communities’ hypothesis, which explores whether barriers to research utilisation are mainly driven by cultural or institutional differences between researchers and decision-makers, and those drawing on Carol Weiss’s typology of ways of using evidence [36,37]. Policy and academic actors were conceptualised as opposing sets of actors, with different priorities, languages, practices, and priorities. For proponents of this perspective, ‘bridging’ this gap becomes a priority. It seems likely, however, that insisting on the existence of this ‘gap’ may polarize previously neutral actors; and indeed this debate fails to recognise that this may be a UK-specific problem (cf. to Dutch studies).

Models of the research and policy processes are, as noted by previous theorists of EBP, rarely made explicit in evaluations of applied research. Where implicit, a simple ‘pipeline’ model is usually assumed (i.e., that the more research is carried out and the higher the quality, the bigger the effect on policy and practice) [38-41].

A large new theoretical strand within health policy focuses on knowledge brokerage/translation as a framework for understanding use of evidence [42-46]. This model can be seen as an extension of Weiss’s ‘instrumental use’ model, or ‘enlightening’ and ‘strategic use’ of evidence, describing the influence of research on policy, and is linked to ideas about ‘coproduction’ and ‘user involvement’ [47].

Weiss and Caplan are still major influences on the field of EBP research, but the degree to which these contributions are appropriately exploited is debatable. The linear (direct use) model is usually perceived to be superior, quite contrary to Weiss’ argument that policymakers thought enlightening (indirect) use could offer more. This message is often overlooked by research (and researchers) in the field, namely that researchers need to reflect on the common view that policymakers are interest-oriented and indifferent to evidence. Barriers to use of research are equated with barriers to direct use of research, while the broader concept of enlightening (indirect) use is rarely seen as equally relevant and useful.

While this is still widespread, theoretical learning from other fields is filtering into the debate. Researchers in policy studies have long seen the policy process itself as a contested arena of negotiation [48,49]. The messy, complex, and serendipitous nature of policymaking is described by Kingdon [50], Weiss [51], Simon [52], and Lindblom [53], amongst others. These scholars have contributed ideas such as punctuated equilibrium [54], policy ‘windows’ and ‘streams’ [50] or ‘stages’ [48], bounded rationality [55], and incrementalism [53]. However, the degree to which these models are used in planning and conducting empirical research is debateable. As Lomas notes, one reason why these may have been resisted by EBP researchers is because they offer little help and no tools to help the aspiring policy-advisor [56].

Of course, many of these are theories about policy, rather than analysis tools to assist advisors [57]. Cairney argues that policy analysts tend to use concepts, such as the policy cycle, which have been rejected by policy scholars. We would qualify this statement by restricting the rejection of such theories to within science and technology studies and policy sciences – these ideas are still common currency within health policy amongst other fields [58].

The influence of these ideas on the health field is perhaps increasing, with applications of ethnographic methods [59] and actor-network theory applied to EBP [60]. However, the majority of studies still use over-simple theoretical models [61]. Asserting the existence of and describing and prescribing interventions to close the ‘research-policy gap’ is a stance which, we argue, is likely to perpetuate and even create gaps between the professions.

## Focus of the research

The main focus of much theoretical and empirical work in EBP has traditionally been, implicitly or explicitly, on research evidence uptake, primarily peer-reviewed research carried out by university-based academics [30,31]. However, a third of studies included in our review examined non-research data, for example, public health surveillance data, strategic needs assessments and other impact assessments, geographic information systems [62], or other non-research evidence [63-65]. This suggests that policymakers interpret and use ‘evidence’ in a broad sense, which is usually not acknowledged by academic commentators [7,10].

The reviews described above included a minority of studies which aimed to describe the policy process in detail, often using a case-study approach. Frequently, these focused on the use of a particular type of evidence such as economic evaluations [66] or the role of a specific piece of evidence [60,67]. Few attempt a descriptive contextualisation or ethnographic understanding of the policy process, with exceptions, e.g., [68-71]. Virginia Berridge’s seminal work on the NHS and comparative health policy development uses historical methods to understand these processes and develop theory around them [72]. Others have taken empirical ethnographic approaches to understand, for instance, how health decision-makers conceptualise and use evidence [73,74]. With these exceptions, very few studies have, as yet, taken anthropological or historical approaches to understanding the role of evidence or research in policy.

Research in the area thus focuses primarily on how to increase uptake of research, on designing and evaluating interventions aiming to increase research use, and on identifying barriers and facilitators of research use by policymakers [74-76]. This pattern of research on evidence use skews the debate by focusing on exceptional cases of research use in policy-making, rather than the normal discharging of statutory business. As Kogan and Henkel noted, attempts to improve use of evidence can “*fail to note how in those areas of policy where data are diffuse, and analyses most likely to be strongly influenced by value preferences, problems must be identified collaboratively between policy-maker and scientist. It failed to acknowledge that policy makers have to work hard to identify problems, to specify research that might help solve them, and to receive and use the results of research*” [11]. While a comment evaluating the Rothschild experiment, the suggestion that EBP research does not reflect the range of knowledge-producing or policy-making processes can also be levied at much of the academic work subsequent to this important study.

Focusing on the use of research evidence also allows researchers to sidestep the rest of the policy process and avoid the context of decision-making more widely. Most studies focus on single elements of the policy-making process – dissemination of evidence, sources, and types of source, knowledge transfer, and priority setting – rather than trying to characterise the process as a whole. EBP research could draw here on an extensive research literature in policy analysis from political science, starting with studies of what is called ‘the policy cycle’ or ‘a stages approach’ [39,77]. Such studies can analyse who is making which decisions, about what, and when; the distinction between practice, management, governance, and policy is rarely spelt out [78]. Clear definitions of policy, use of research, and decision making has been encouraged for over 60 years, but remains evasive [79].

## Methods

Considerable theoretical work has gone into producing taxonomies of factors influencing the utilisation of evidence, e.g., [36,80]. However, these rich theoretical discourses are not reflected in the bulk of the empirical literature, which, despite its breadth with respect to the sectors and categories of evidence types studied, finds fairly consistently that the main factors affecting use of evidence are (a) access to relevant and clear information and (b) good relationships between researchers and research users. This may be due to the methods used in the studies: most use only interviews or surveys to ask researchers and policy-makers about their perceptions about evidence use; very few use methods such as participant observation to observe how evidence is actually used in practice, or attempt to find documentary proof of research use (with exceptions; [76]). These lists, while important, cannot on their own lead us to an improved understanding of the role of evidence in the jigsaw of the policy process.

Another noticeable feature of the evidence base is the emphasis given to researchers’ own views of research utilisation, for example, a study aiming to look at everyday working practice, such as that of Taylor-Robinson et al. [81], samples primarily health inequalities/public health academics and practitioners rather than decision-makers themselves (such as councillors or executives), even though they themselves describe lack of contact between academics and policymakers as a barrier to use of evidence. The majority of academic studies in EBP research are, unsurprisingly, written by and for academics, with little involvement of policy-makers as co-authors; indicating that policy-makers are not involved in developing or carrying out relevant research.

## Underlying assumptions of this research and critical reflections

This overview of the literature provides a starting point for a more critical engagement with the empirical literature on EBP. We provide a broad-brush characterisation of parts of the literature which helps to draw out common assumptions across the field as a whole and enable critical reflection on them. We focus on three such assumptions: 1) that the policy-evidence ‘gap’ needs ‘bridging’; 2) that policy is usually not based on any data, and policy requires research evidence, preferably evaluative intervention research; and 3) that greater use of evidence in policy-making will produce better outcomes at a population level. It is by no means the case that these assumptions are universally shared among EBP researchers, or that we are the first to identify these issues [61,82,83]. Nonetheless, a large proportion of the available research still rests on an uncritical acceptance of these assumptions. Below, we describe the effect of these assumptions, justify our rejection of them, and discuss the implications of taking a more critical approach.

## Assumption 1: that a policy-evidence ‘gap’ exists

The ‘evidence-policy gap’ is a widely-acknowledged construction in policy-related research, asserting the existence of two separate communities with their own ecosystems and languages [61,83-85]. Much of the ‘knowledge translation’ literature, which attempts to take a broader perspective on EBP, fails to question the assumption that knowledge and practice are two separate practices, and the ‘joining’ or ‘bridging’ of these (depending on the authors’ preferred metaphor) is the task of EBP researchers; see, e.g., [20,61,83,84,86]. However, as Choi recognizes, evidence – and those associated with evidence – is just one voice among many [87]. We do not yet know how to make that voice more helpful nor more influential.

Recently, a shift away from this dichotomous debate has been made, with concepts such as ‘knowledge translation’ or ‘transfer’ being replaced by ideas about ‘learning’, ‘contribution’, and co-production [88]. These ideas frame the relationship between research and policy as a two-way negotiation in which both partners learn from the other – pragmatically and politically a step towards an equality of prioritisation and experience. Certainly, this represents a greater openness to seeing a broader range of data types as relevant – including, for example, contextual, descriptive data as well as evaluations and other forms of research evidence. Too often, however, this debate is hijacked by methodologists from opposing camps wishing to defend their own method in the face of criticism – whether real or perceived, e.g., [89,90]. Without clear definitions of ‘evidence’ and ‘impact’ or ‘learning’, these studies contribute to negative stereotypes on both sides, and perpetuate the gap they aim to bridge.

## Assumption 2: that policy is usually not based on evidence

Despite the increased literature in the area, there is a surprising lack of evidence about how much evidence policymakers use. Studies have largely reported policymakers’ perceptions of their usage (e.g., [91]), acknowledge that it is impossible to tell how much evidence was used by policy participants [92], or rely on self-reported measures [93]. EBP researchers have tended to interpret this absence of any contradictory evidence as a confirmation of their belief that policymakers do not use evidence. This is both grossly unfair to policymakers who have been shown to draw on a wide range of information sources [39], hugely over-simplifies the relationship between evidence and policy, and, of course, contradicts the avowed principles of EBP researchers; viz, that beliefs ought to be based on evidence [76].

Implicit in the ‘barriers and facilitators’ approach is an assumption that if these factors were alleviated, research uptake would increase. However, this is to miss the key point, which is that most research in the area studies the use of research evidence by policymakers, not what knowledge or information policymakers use. This subtle shift in emphasis opens up new avenues of enquiry. Other information than research evidence might be more relevant and timely, two factors seen as top facilitators for policymakers’ use of evidence [94]. Policymakers may prefer to use local information or intelligence such as patient or practice level data, or that held by local councils (e.g., datasets of rent, crime, and transport) [95]. It seems likely that these sources of information have been undervalued by evaluation methodologists, who often value trial data above other types. A more naturalistic approach using empirical methods to study policymakers *in vivo*, would conceive of evidence as one of many influences on a decision.

## Assumption 3: that use of more research evidence by policymakers would lead to ‘better’ policy

We are not the first to note that “[t]*he assumption that the use of evidence would improve the outcome of the policy process remains relatively untested by any form of empirical analysis*” [96-98]. The absence of robust evaluation evidence showing that evidence utilisation actually leads to better outcomes is widely admitted. The bulk of the intervention evidence in EBP uses only research utilisation or uptake as an outcome (or, in some cases, merely attitudes and intentions regarding research use). Nonetheless, it is still widely claimed that decisions made in partnership between “*politicians and researchers & lay people are more likely to result in positive health outcomes*” [86] and many researchers continue to advocate for increased use of research evidence [99].

Such claims, where they are not treated as automatically self-evident, are usually supported either by anecdotal cases of increased evidence use leading to better outcomes, or by studies of the impacts of evidence use on process measures such as transparency of decision-making [100]. However, the value of process-oriented goals is surely questionable, if they cannot be shown to lead to improved health, wellbeing, social, or other outcomes for the putative beneficiaries of the policy in question. The typologies of ‘research impact’ which have dominated much work in this area (e.g., [101]) are of limited value without a more open debate about the correct metrics for evaluating EBP, based on a realistic view of the currently existing evidence base [102]. Moreover, much of the commentary around the ‘impact agenda’ focuses on the aspect of academic performance management, without wider examination of its connection to policy and knowledge practices and theories [16].

Even in the absence of robust evaluation data, it is clear that many of the existing theoretical rationales for how evidence utilisation is supposed to improve outcomes are inadequate. If the pipeline model of research use were correct, it would be possible to demonstrate the impact of research on policy and the value of research would be judged on its contribution to policy and its quantifiable impact [25]. This model “*fails the practitioner because the literature on which guidelines are based constitutes an unrepresentative sample of the varied circumstances and populations in which the intervention might be usable or unusable*” [38].

## New directions for EBP research

Above, we describe three assumptions commonly found in the EBP literature. We critically discuss the reasons we believe these assumptions are flawed, and show how the existing research conducted on the basis of these assumptions is likely to fail to answer the most pressing problems in EBP. Here, we describe how these assumptions can form the basis for a new programme of research aiming to understand the relationship between science and policy. We explore the implications of re-framing future studies in a new direction, and suggest more explorative perspectives and participative methods.

Firstly, the assumption that the policy-evidence ‘gap’ needs ‘bridging’. Most of the studies identified perpetuate the division between the ‘two communities’ in one way or another. Approaching researchers and policy-makers separately and asking them for their accounts of evidence use may be likely to produce these conflicting accounts; similarly, asking researchers about their perceptions of what policy-makers do may not be the most sensitive way of exploring policy processes. It would be more interesting, and more novel, to approach policymakers from an unprejudiced stance, to describe their activities, and to identify how they populate policy areas and steer policies through [53]. Of course, we are not the first to suggest this [56,97] – but these studies are generally the exception rather than the rule.

Secondly, the assumption is often held that policy is usually not based on any data, and policy requires research evidence, preferably evaluative intervention research. By concerning themselves with questions such as “*how* [can] *the tension between scientific rigour and timely relevance to policy-making be handled*” [103], EBP researchers often fail to acknowledge their lack of knowledge about forms and models of the impact and contributions of evidence to policy processes, which can lead to the creation of unhelpful straw men.

Finally, the unspoken corollary to both these assumptions is that greater use of evidence in policy-making will produce ‘better policy’ and better outcomes at a population level. Leaving aside the question of what constitutes ‘better’ in a self-evidently political and therefore value-driven terrain, for researchers to convincingly argue for the increased use of evidence in policy making, they must be able to demonstrate the benefits of doing so. The growth of the ‘applied research’ sector claims to address this, but often restricts output to vague and untested policy and research recommendations, about which there is no evidence of effectiveness [99]. If the effects of these policy and research recommendations are not evaluated these could be misguided at best [104].

Therefore, we argue that the following issues are of outstanding importance and could form the basis for a new agenda of EBP research:

1. Refocus research on influences on and processes of policy rather than how to increase the amount of evidence used. Researchers in political and policy studies, anthropology and history of policy, and science and technology studies have provided a wealth of insights and rich empirical data on the functioning (or otherwise) of the policy-making process [13,53,56,68-70,88,105-108]. Understanding the daily lives and activities of policy actors can bring fresh insights into how ‘evidence’ is conceptualised, the potential roles it may play, and how it fits with the other drivers and triggers which affect policy [95,109]. Understanding the roles of exceptional individuals, such as policy entrepreneurs, and networks in the policy process is also recognised as a key research area [3,110,111].

Dialogue between these fields has not been very extensive or productive so far, largely due to scepticism on the part of policy scientists, e.g., [108], and a lack of engagement with this body of theory and empirical data, with recent exceptions [112,113]. To take only one example, policy researchers observe that policies rarely have a consistent and well-defined goal or aim; rather, “*solutions become joined to problems*” in a provisional and largely haphazard way, with the same policy taking on different goals at different times or in different contexts [50,114]. If this is the case, the question of how researchers can evaluate whether or not the policy has attained its goal, and use the results of this evaluation to inform future policy development, is largely moot.

2. Determine what information and evidence is normally used as part of policy processes. As we point out above, it is likely that the needs and practices of policymakers are rarely the subject of rigorous study, and are certainly more complex and nuanced than can be captured in surveys. Using ‘research’ and ‘policy’ as one-size-fits-all concepts underestimates the variety of activities and outputs involved in each type of process. A legislative manoeuvre is very different from a local tailoring of licensing hours; applied health research aiming to develop interventions about a specific condition is worlds away from contemplative studies of models of theories of change. Elucidation of the relationships at both ends of the spectra ought to be a research priority, in place of the dichotomising of activities as ‘research’ or ‘policy’. Furthermore, attention to context is vital; pressures faced by researchers and policymakers in low- and middle-income countries may be very different from those in Western settings. Finally, as argued above, although similar barriers and facilitators are often identified across policy settings [35], there is variation of practices and processes across policy areas. Analysis of these variations would be a whole research agenda in itself. A subtler interpretation of context, ‘policy’, and ‘research’ are necessary to understand processes, influences and impacts, and indeed to develop any meaningful abstractions and generalisations – should these be possible, which is by no means certain. A compromise between the complexity of policy making and development of useful frameworks needs to be found.

3. These questions are likely to require a broader range of methodologies than usually applied. Experimental, ethnographic, and conceptual studies all need to be applied to understand the impact of evidence on policy and policy processes more generally. Novel theoretical approaches could be phenomenological, psychosocial, or interpretive [115-117].

4. Develop conceptual clarity around and metrics to evaluate ‘impact’ of research on policy and populations. Without clear methods to understand how policy works and how it changes in response to information, it will be impossible for researchers to know whether they have had an effect on policy. Research usage could take many forms, from agenda-setting, to provision of policy options, to challenging debates, or refuting arguments. Impact could therefore be a change in policy, consistency in policy, changes in population level outcomes. Measures proposed thus far tend to focus on citations or mentions of work by policymakers. We feel this addresses only a narrow aspect of potential impacts, namely awareness of research, which may not translate into action. Furthermore, this metric-led approach tends to ignore the indirect means by which research and evidence of all kinds fit into policy – whether by sustaining the status quo, or by leading to decisions for change. More attention to the variety of impacts and effects leading from research may help to develop this debate.

5. Finally, our analysis of the literature suggests that new methods and organisations aiming to bring the processes of research and policy closer together are likely to further our understanding of the relationship between these two types of activities. Co-creation and co-production of knowledge are lauded as a more democratic, and potentially more useful, type of learning activity than many other knowledge exchange events [118]. If universities were to provide assistance for local policymakers in the analysis of existing data, a relationship of mutual benefit could start to develop – an end in itself, according to reviews of barriers and facilitators of evidence use [30,34]. Moreover, such organizations would provide natural laboratories for studying the role of institutional and organizational factors on the practices of policy formation and implementation, noted in our review as likely to affect evidence use.

Perhaps due to the political, financial, and ethical pressures on health policymakers to make good decisions, health policy leads the way in forming collaborative organisations to conduct research. Funders in the UK and Netherlands have developed specific types of collaborative engagement organisations (e.g., Collaborations for Leadership in Applied Health Research and Care, CLARHCs, or the health funder ZonMw), which aim to bring practitioners and researchers together for mutual benefit. There are examples of institutions which specifically allow researchers and policymakers to learn about each other’s priorities and ways of working [56,119]. In general, however, the literature above suggests that there are insufficient opportunities and incentives to form links with policymakers directly.

## Conclusions

The existing literature on EBP has certainly contributed to the desirable outcome of better policy decisions and acceptance that evidence ought to play a role in those decisions. Now, we believe it is time for researchers to reflect on their assumptions, develop new perspectives, and use other methods to tackle the problems of EBP. There is a common lack of clarity about what researchers understand as ‘policy’, which can encompass decision making, project implementation and evaluation, and service reconfiguration. There is, in general, little evidence about management and organisation, despite these being potentially major factors affecting the policy process [120]. The assumptions governing the design and outcomes of research into policy will probably be significantly different in different institutional areas. It is often not clear what constitutes ‘a decision’, nor who is involved in it, or whether research evidence is relevant or timely. This muddles and prevents any engagement with discussions about what constitutes good and bad policy; or indeed how evidence ought to be used.

Rather than attempting to develop a one-size-fits-all (pipeline) model, research in this area should revert to observational methods with in-depth descriptions of practices and their inherited processes and provide an empirical basis for theoretical development which can inform future activities. Instead of repeating studies of perceptions of barriers and facilitators of use of research evidence, appropriate methods must be used to answer questions about when, why, how, and who finds what type of knowledge sound, timely, and relevant at different stages of the policy cycle.

The position of researchers who wish to influence policy is untenable unless there is engagement with the questions outlined above. We would also argue that researchers who advocate for change (for example, via policy and practice recommendations) without evaluating the (likely) impact of these changes may have a limited effect at best. At worst, they create distance between policy and research by demonstrating an understanding of the context within which they would like their newly-generated knowledge to be used. Without understanding the complex processes of policy and knowledge mobilisation, researchers who make policy and practice recommendations may simply be ignored. Ultimately, the role of researchers is not to judge the ‘quality’ of policy making on the basis of how much of their research is used. This stance is both unhelpful and divisive, blinding researchers to the important questions raised above regarding the types of information used, by whom, for what purpose, and under which circumstances. Rather, our role as scientists ought to be to investigate the processes surrounding the use of evidence and policy activities more widely and to disseminate findings in order to help others make informed decisions, of all kinds.

## Abbreviations

EBM: Evidence based medicine; EBP: Evidence based policy.

## Competing interests

The authors declare no competing interests. This study was not supported by any funding agency.

## Authors’ contributions

All authors contributed to the conception, design, and analysis of the data which underpins this critical review. All authors contributed to the development of the argument and the final manuscript. All authors read and approved the final manuscript.
